# Development of package payment based on UNU-CBGs Casemix system for provider payment in Aceh Health Insurance, Indonesia

**DOI:** 10.1186/1472-6963-12-S1-O8

**Published:** 2012-11-21

**Authors:** Irwan Saputra, Syed Mohamed Aljunid, Amrizal Muhammad Nur

**Affiliations:** 1 United Nations University International Institute for Global Health (UNU-IIGH), Kuala Lumpur, Malaysia

## Background

The Aceh Health Insurance (JKA) has been established since June 1, 2010. Presently, the provincial Government of Aceh faces difficulty in getting adequate budget to fund JKA program that tends to increase every year. In the year 2010, the second year of JKA implementation, the government was only able to allocate Rp. 382 billion from the total requirement of Rp. 482 billion. The figure will possibly continue to increase next year because of the increase in the overall hospital tariff of adjustments where there was an increase over 50%. In addition, there were complaints that quality of care is not optimum. The fee for service reimbursement system in JKA is one of the reasons for increase in cost of JKA. The main purposes of this study are to develop and implement the Casemix system as a provider payment mechanism and to assess its feasibilities to improve the performance of JKA program.

## Method

Qualitative approach using in-depth interview will be conducted in this research to evaluate the current reimbursement system of JKA. Major stakeholders in the JKA program such as governor, representative council, and head of health directors of hospital will be sampled in this study. Quantitative methods will be applied to collect the data for Casemix implementation from selected hospitals on the data of disease coding and hospital costing. Costing data will be analyzed using Clinical Cost Modeling (CCM) software, whereas the coding data use UNU-CBGs grouper software to develop new hospital tariff for JKA.

## Result

The expected result of this research is on the evaluation of JKA implementation. Simulation exercises will be used to compare with the current reimbursement system and to find out the impact of the newly developed tariff. At the end of this study, the researcher will come up with some recommendations on the use of Casemix system for JKA program.

## Conclusion

This study will provide valuable input to enhance the implementation of JKA in Aceh. The outcome of the study will develop new Casemix based tariff which has positive impact on the sustainability of JKA program.

